# Simulating Cancer: Computational Models in Oncology

**DOI:** 10.3389/fonc.2013.00233

**Published:** 2013-09-13

**Authors:** Heiko Enderling, Katarzyna Anna Rejniak

**Affiliations:** ^1^Center of Cancer Systems Biology, St. Elizabeth's Medical Center, Tufts University School of MedicineBoston, MA, USA; ^2^Integrated Mathematical Oncology, H. Lee Moffitt Cancer Center & Research InstituteTampa, FL, USA; ^3^Department of Oncologic Sciences, College of Medicine, University of South FloridaTampa, FL, USA

**Keywords:** computational modeling, computational oncology, tumor development, tumor treatment, *in silico* cancer

Cancer is one of the deadliest diseases of our time. While the war on cancer has cost many billions of dollars, the mechanisms underlying tumor development, progression, and therapeutic cure or control are yet to be fully understood. An interdisciplinary effort that brings together clinicians and biologists with mathematical and computational modelers is therefore necessary. Mathematical modeling and computational simulations bring to the table sophisticated tools for analyzing experimental data as well as for systematic, quantitative, and multi-scale *in silico* experimentation. Taken together, such an interdisciplinary approach promises to shed light on the underlying rules of the intra-, inter-, and extracellular mechanisms behind complex tumor dynamics, with the ultimate aim to predict patient-specific treatment outcomes.

The papers in this Special Topic span a broad spectrum of cancer cell-related subjects from intracellular modifications in individual cells to complex interactions between tumor cells and tumor microenvironments to emerging behaviors of cell populations on the organ and whole body scale. Quantitative modeling has been applied to virtually every type of tumor. This collection includes papers on brain, ovarian, and colon cancers, as well as on melanomas, leukemias, sarcomas, and head and neck tumors. The models also addressed various stages of tumor development including its initiation, growth, invasion of the surrounding stroma, tumor cell migration, and intravascular transport, as well as metastatic colonization. Various types of anticancer treatments have been discussed in this Special Topic, including chemotherapy, radiotherapy, immunotherapy, and differentiation therapy. From a mathematical point of view, the models range from deterministic to stochastic, from continuous population dynamics to agent-based individual cell models, from fluid dynamics to Monte Carlo simulations and energy minimization models.

We divided the papers in this Special Topic into five categories. In the first, the subcellular mechanisms and their impact on a single cell and population-level heterogeneity are considered. Dynamics on the subcellular scale include intracellular gene modulations or extracellular diffusion of soluble factors. Leenders and Tuszynski (1) discuss both stochastic and deterministic models of p53 protein regulation that play a crucial role in cellular stress and DNA damage response. Kimmel and Corey (2) show that large variations in the timing of transitions from neutropenia to acute myeloid leukemia can be explained by stochasticity in cell driver mutations. Howk et al. (3) use a single cell model of two-hit mutations of normal cells into endometrial cancer cells to predict the frequency of cancer stem cells in endometrial cancer. Jain and Jackson (4) present a hybrid model that simulates the dynamics of vascular endothelial growth factor (VEGF) diffusion and its binding to endothelial cell receptors, which triggers endothelial cell activation and polarization during angiogenesis. Finley et al. (5) consider another angiogenesis model centered on VEGF and its molecular interactions that has been calibrated to experimental data and shows that *in vivo* VEGF secretion rates are significantly lower than most reported *in vitro* measurements, which has profound implications for anti-angiogenic treatment.

The second class of papers contains mechanical models of tumor invasion that are influenced by both the physical forces and chemical factors necessary to degrade the host tissue. Deakin and Chaplain (6) present a mathematical model focusing on both soluble and membrane-bound metalloproteinases (MMPs) and their relative role in the degradation of highly dense collagen structures and cross-linked fibers. Mumenthaler et al. (7) discuss an integrative experimental–computational approach to understand MMP-mediated tissue degradation. The fluid-generated forces exerted on the cell either by the interstitial fluid or shear stress in the blood circulation are reviewed by Mitchell and King (8) from both experimental and computational perspectives. Katira et al. (9) discuss interdependence of mechanical and biological pathways within the cell and how intracellular and environmental mechanical properties, such as stiffness and adhesivity, lead to changes in cell behavior, including transformation into malignancy. Wallace and Guo (10) analyze mathematical models of avascular tumor growth and conditions under which the models reproduce the growth dynamics of *in vitro* spheroids.

The third group of studies investigates cancer stem cells. With experimental stem cell purification and reliable identification still in its infancy, mathematical models highlight the population-level dynamics resulting from different stem cell kinetics. Rodriguez-Brenes et al. (11) discuss homeostasis in stem cell lineages through tightly controlled feedback mechanisms that regulate stem cell proliferation and self-renewal. Enderling et al. (12) examine how tumors grow if the cancer population is fueled by a cancer stem cell, showing that tumors exhibit a variety of irregular morphologies and harbor stem cell fractions that vary by many orders of magnitude and evolve over time. Bachman and Hillen (13) investigate how conventional radiotherapy can be complemented by differentiation therapy that forces stem cells into differentiation to increase their sensitivity to radiation.

The next group of articles focuses on interactions between the tumor and its microenvironment. Szabó and Merks (14) discuss avascular and vascular tumor growth and evolution using the Cellular Potts model. Orlando et al. (15) examine tumor cell evolution at the tumor core and its invasive edge, focusing on the effects of colonization tradeoffs on tumor invasion dynamics. Steinkamp et al. (16) integrate *in vivo* xenograft mouse models and mathematical models to study tumor attachment, invasion, and vascularization in the ovary, showing that local factors and mesothelial lining features strongly influence invasion.

The final group of studies focuses on the use of quantitative models to improve treatment modalities. Patient-specific mathematical neuro-oncology approaches are reviewed by Baldock et al. (17). Kim (18) presents a mathematical model based on microRNAs that balance cell proliferation and migration in different microenvironmental conditions in glioblastoma, suggesting a post-surgery injection of chemoattractants and glucose to counteract the diffusive spread of residual cells. Hawkins-Daarud et al. (19) discuss a model of fluid accumulation in gliomas during anti-angiogenic therapy and discuss the implications of the environmental response to tumor growth on medical imaging. Rejniak et al. (20) present an integrative study examining penetration and efficacy of therapeutic agents in relation to tumor tissue architecture. DePillis et al. (21) use a model of dendritic cell therapy on melanoma, showing how dosage and schedule modifications enhance immunotherapy efficacy.

The images featured on page 2 of this e-book showcase computational models discussed in detail in this Special Topic. Clockwise from top left: a schematic of miR-451 activity in the model of Kim (18); a 2-D slice through the ovarian tumor simulated using the Potts model of Steinkamp et al. (16); a 3-D simulation of malignant glioma cells from Baldock et al. (17); cancer stem cell-driven tumor growth from Enderling et al. (12).
